# Associations between subjective sleep quality and inflammatory markers in patients with treatment-resistant depression

**DOI:** 10.1017/S1092852925000227

**Published:** 2025-04-11

**Authors:** Mao-Hsuan Huang, Mu-Hong Chen, Pei-Chi Tu, Ya Mei Bai, Tung-Ping Su, Yee-Lam E Chan, Cheng-Ta Li

**Affiliations:** 1 Department of Psychiatry, Cheng Hsin General Hospital, Taipei, Taiwan; 2 Division of Psychiatry, Faculty of Medicine, National Yang Ming Chiao Tung University, Taipei, Taiwan; 3 Institute of Brain Science and Brain Research Center, School of Medicine, National Yang Ming Chiao Tung University, Taipei, Taiwan; 4 Department of Psychiatry, Taipei Veterans General Hospital, Taipei, Taiwan; 5 Department of Medical Research, Taipei Veterans General Hospital, Taipei, Taiwan

**Keywords:** Depression, inflammation, insomnia, sleep quality, tumor necrosis factor

## Abstract

**Background:**

Sleep disturbances are prevalent in major depressive disorder (MDD). Emerging evidence suggests a bidirectional relationship between inflammation and sleep disturbances, but the role of peripheral inflammatory markers in subjective sleep quality in treatment-resistant depression (TRD) remains unclear.

**Methods:**

34 MDD patients (20 TRD and 14 non-TRD) and 34 healthy controls were enrolled. Participants underwent clinical assessments, including the Hamilton Rating Scale for Depression and Pittsburgh Sleep Quality Index (PSQI). Serum levels of inflammatory markers, including soluble interleukin-2 receptor (sIL-2R), soluble interleukin-6 receptor, soluble tumor necrosis factor-α receptor type 1 (sTNF-αR1), monocyte chemoattractant protein-1, and C-reactive protein, were measured. General linear models were used to assess associations between inflammatory markers and subjective sleep quality, adjusting for relevant covariates.

**Results:**

Patients with MDD scored higher in PSQI than healthy subjects. Higher serum levels of sTNF-αR1 were associated with longer sleep latency across the TRD and non-TRD groups. Elevated serum sIL-2R levels correlated with poorer overall sleep quality among patients with MDD.

**Conclusions:**

These findings underscored the importance of considering inflammatory pathways in understanding sleep disturbances in depression. Longitudinal studies are needed to elucidate causal relationships and inform potential therapeutic interventions targeting both inflammation and sleep in MDD.

## Highlights

1. Patients with depression had poorer subjective sleep quality than healthy subjects.
2. Serum sTNF-αR1 positively correlated with sleep latency in treatment-resistant depression.
3. Serum sIL-2R negatively associated with overall sleep quality in depression.

## Introduction

Sleep disturbances represent a prevalent manifestation of major depressive disorder (MDD), with over 75% of afflicted individuals reporting significant disruptions in sleep patterns.^1^ Patients with MDD exhibit decreased sleep efficiency, increased nocturnal awakenings, reduced latency to rapid eye movement (REM) sleep, and increased REM density compared to healthy controls.^2^ Notably, sleep disturbances not only serve as a primary risk factor for depression but also correlate with diminished quality of life, heightened suicide risk, and compromised response to conventional antidepressant therapies.^1^ Inflammation emerges as a potential pathophysiological mechanism underlying sleep disturbances in depression, supported by accumulating evidence suggesting that activation of immune-inflammatory pathways contributes significantly to the development of MDD and may be relevant to its pathogenesis in specific subpopulations. Inflammatory markers, including acute phase proteins, cytokines, and their receptors, were found to access the central nervous system (CNS), thereby interfering with key physiological processes, including neuroplasticity, neurotransmitter metabolism, neuroendocrine function, and neuronal apoptosis.^3^ Inflammatory markers associated with depression include C-reactive protein (CRP), interleukin-2 (IL-2), interleukin-6 (IL-6), tumor necrosis factor alpha (TNF-α), and monocyte chemotactic protein-1 (MCP-1).^4^^-^^6^ Bidirectional causative mechanisms intertwining inflammation and sleep disturbances have been posited,^7^ with cytokines such as IL-6 and TNF-α potentially affecting brain regions involved in sleep regulation, consequently altering sleep architecture.^8^^,^^9^ Preclinical models administering inflammatory cytokines to cancer patients have demonstrated diminished sleep quality and duration.^10^ Conversely, autonomic nervous system activation and heightened catecholamine levels during sleep deprivation may stimulate the production of inflammatory markers.^11^ Meta-analyses from cohort studies and sleep deprivation experiments have indicated a link between sleep disturbances and elevated systemic inflammatory markers: IL-6 and CRP.^12^ However, discrepancies exist within the literature, as certain investigations observed no associations between insomnia symptoms and IL-6.^13^^,^^14^ The variance in findings may stem from inconsistent insomnia symptom assessment methodologies and the inclusion of participants with diverse diagnoses. Additionally, scant attention has been paid to mood symptoms frequently co-occurring with poor sleep, such as depression, potentially contributing to the inconsistent findings regarding insomnia and inflammation.^15^

Treatment-resistant depression (TRD) represents a subgroup of individuals with MDD who do not experience significant improvement in symptoms despite trying multiple standard antidepressant treatments, leading to chronic, severe, and debilitating symptoms that impair daily functioning and quality of life.^16^ While inflammation may contribute to the pathophysiology of depression, TRD was shown to be associated more with immune activation than with treatment-responsive depression,^17^^,^^18^ and pro-inflammatory cytokines have been demonstrated to be a therapeutic target for patients with TRD.^19^ Investigating the relationship between sleep disturbance and inflammation among patients with MDD, especially TRD, is crucial for advancing our understanding of depression, identifying potential treatment targets, and enhancing clinical care. In a previous study, we examined peripheral levels of pro-inflammatory cytokines in MDD and found a higher serum concentration of soluble tumor necrosis factor-α receptor type 1 (sTNF-αR1) among patients with TRD compared to non-TRD patients and healthy subjects.^20^ In this paper, we extended our analysis to examine the role of peripheral inflammatory markers in subjective sleep quality among patients with TRD and treatment-responsive depression, utilizing the same dataset. This allows us to build upon our earlier findings and provide a more comprehensive understanding of how inflammation might contribute to adverse health outcomes in depression. We hypothesized that elevated peripheral pro-inflammatory markers would be associated with poor subjective sleep quality among patients with MDD.

## Methods

34 consecutive outpatients, aged 25 to 65 years, diagnosed with MDD using the Mini-International Neuropsychiatric Interview (MINI) and the Diagnostic and Statistical Manual of Mental Disorders-IV-Text Revision (DSM-IV-TR), were recruited from the psychiatric outpatient department of Taipei Veterans General Hospital. Among these individuals, 20 had a documented history of resistance to antidepressants (TRD group), while 14 did not (non-TRD group). To confirm antidepressant resistance, we adhered to established criteria (failure to respond to at least 2 different classes of antidepressant trials with adequate dosage and duration in the current or past episode). Exclusion criteria encompassed significant physical illness, alcohol or substance use disorder history, and major psychiatric comorbidities such as schizophrenia, bipolar disorder, or other psychotic disorders. Additionally, 34 healthy individuals, matched for age and sex, underwent the MINI assessment with a psychiatrist to ensure the absence of psychiatric illness. A comprehensive medical history review was conducted to rule out physical illness in all participants. The study adhered to the Declaration of Helsinki guidelines and received approval from the Institutional Review Board of Taipei Veterans General Hospital (V102B-033), with all participants providing written informed consent.

## Clinical assessment

Demographic characteristics, encompassing age, sex, body mass index (BMI), and psychotropic medication use, were documented for each participant. Mood severity evaluations were conducted individually by an experienced psychiatrist, incorporating assessments derived from the 17-item Hamilton Rating Scale for Depression (HAMD-17).

Subjective sleep quality was evaluated using the Pittsburgh Sleep Quality Index (PSQI), a self-administered questionnaire designed to assess sleep quality over the preceding month. The PSQI encompasses 7 dimensions of sleep, namely component 1: subjective sleep quality, component 2: sleep latency, component 3: sleep duration, component 4: sleep efficiency, component 5: sleep disturbances, component 6: use of hypnotic medications, and component 7: daytime dysfunction. Each dimension is rated from 0 to 3, with higher scores suggesting poorer sleep quality. The aggregate of these scores yields the total PSQI score, ranging from 0 to 21. The PSQI is a validated tool, with reliability established against polysomnography, the gold standard for sleep measurement.^21^ In this study, a PSQI total score > 5 indicates poor sleepers, and a total score ≥ 11 is indicative of severe sleep disturbance.^22^

## Laboratory measurement

IL-6 demonstrates a multifaceted impact, both neuroprotective and neurodegenerative, on inflammation and immune response within the CNS.^23^ This cytokine exerts its biological influence through classical signaling and trans-signaling mechanisms, facilitated by its binding to both the IL-6 receptor and the soluble form of the IL-6 receptor (sIL-6R), respectively. Activation of the IL-6 receptor complex (the binding of IL-6 to IL-6 receptor) prompts the dimerization of the signal-transducing receptor subunit gp130, initiating downstream signaling cascades. While classical signaling exhibits both pro- and anti-inflammatory effects, IL-6 trans-signaling predominantly elicits pro-inflammatory responses.^23^ Evaluation of serum levels of soluble IL-6 receptors may offer superior insights into inflammatory activity compared to IL-6 itself.^24^ TNF-α operates through interaction with 2 receptors, TNF-α receptor subtype 1 (TNF-αR1) and TNF-α receptor subtype 2 (TNF-αR2). TNF-αR1 engagement has been associated with apoptotic neuronal death, whereas TNF-αR2 plays a dominant role in dampening TNF-mediated inflammatory responses.^25^ Soluble forms of TNF-α receptors extend the half-life of TNF-α, with circulating levels serving as indicators of TNF-α production. Consequently, sTNF-αR1 emerges as a more dependable marker of pro-inflammatory activity.^26^ MCP-1, or C-C motif chemokine ligand 2 (CCL2), serves a critical function in inflammation by recruiting monocytes and other leukocytes to sites of inflammation. Beyond recruitment, MCP-1 activation of monocytes can lead to their differentiation into macrophages or dendritic cells, intensifying the inflammatory cascade through the release of additional cytokines.^27^

Enzyme-linked immunosorbent assay (ELISA) kits sourced from R&D Systems, Minneapolis, MN, USA, were utilized to assess pro-inflammatory cytokines, namely CRP, soluble interleukin-6 receptor (sIL-6R), soluble interleukin-2 receptor (sIL-2R), sTNF-αR1, and MCP-1. The lower detection limit of CRP is 0.010 mg/L with a sensitivity of 0.005 ng/mL. Specificity: natural and recombinant human CRP. Cross-reactivity: <0.5% cross-reactivity was observed with available related molecules. <50% cross-species reactivity was observed with the species tested. The lower detection limit of sIL-6R was 6.5 pg/mL, and the sensitivity was 1.5 pg/mL. Specificity: natural and recombinant human sIL-6R. Cross-reactivity: <0.5% cross-reactivity was observed with available related molecules. Cross-species reactivity was not tested. The lower detection limit of sIL-2R was less than 10 pg/mL. Specificity: natural and recombinant human sIL-2R alpha. Cross-reactivity: <0.5% cross-reactivity observed with available related molecules. Cross-species reactivity not tested. The lower detection limit of sTNF-αR1 was 0.77 pg/mL, and the sensitivity was 0.43 pg/mL. Specificity: natural and recombinant human sTNF-αR1. Cross-reactivity: < 0.5% cross-reactivity was observed with available related molecules. < 50% cross-species reactivity was observed with the species tested. The lower detection limit of MCP-1 was 10 pg/mL, and the sensitivity was 1.7 pg/mL. Specificity: natural and recombinant human MCP-1. Cross-reactivity: < 0.5% cross-reactivity observed with available related molecules. < 50% cross-species reactivity observed with species tested. The fasting serum samples were collected in serum separator tubes, clotted for 30 minute, and stored at −80 °C until use. All assays were performed according to the vendor’s instructions. The final absorbance of the mixture was measured and analyzed at 450 nm using an ELISA plate reader with Bio-Tek Power Wave Xs and Bio-Tek’s KC junior software (Winooski, VT, USA). The standard range depended on the vendor’s instructions. A linear regression R-square value of ≥0.95 represented a reliable standard curve.

## Statistical analyses

The assumption of normality for pro-inflammatory cytokine levels was checked using Shapiro–Wilk Test. We found that peripheral levels of sTNF-αR1, sIL-6R, and sIL-2R conformed to the normal distribution (all p > 0.05), while levels of CRP and MCP-1 were positively skewed; therefore, serum levels of pro-inflammatory markers were log-transformed to obtain normally distributed variables. Comparison of continuous variables was performed using Analysis of Variance (ANOVA) (or Student’s t test), while categorical data was evaluated using the chi-squared test. The Levene test was conducted to test differences in variances among groups. In cases of uneven distribution of variance homogeneity, Welch’s one-way analysis of variance was employed, followed by post-hoc pairwise comparisons between groups utilizing the Games-Howell test. Participants were stratified according to their disease, subjective ratings in PSQI total scores (≥11, <11, controls), or ratings in PSQI subscores (≥3, <3, controls). General linear models (GLMs) were used to assess serum levels of pro-inflammatory markers between groups, with the adjustment of age, sex, BMI, HAMD-17 total scores, duration of illness, psychotropic medication use. Linear regression models were used to examine the association between serum levels of pro-inflammatory markers and total/subscores of PSQI while controlling for age, sex, BMI, HAMD-17 total scores, duration of illness, psychotropic medication use, and disease group. For the management of multiple comparisons, the Benjamini–Hochberg false discovery method was employed. Significance was determined at a threshold of p < 0.05. Statistical analysis was performed using SPSS 22 (SPSS Inc., Chicago, IL, USA).

## Results

In all, 34 patients with MDD, including 20 TRD patients and 14 non-TRD patients, and 34 age- and sex-matched healthy subjects were recruited. Both patients with TRD and non-TRD scored higher in PSQI total scores and subscores in sleep quality, sleep latency, sleep disturbances, use of sleeping medications, and daytime dysfunction than healthy individuals (Table 1). The percentage of severe sleep disturbance (PSQI total score ≥ 11) was 70%, 42.9%, and 2.9% among patients with TRD, non-TRD, and controls, respectively.

**Table 1.** Demographic Data, Inflammatory Markers, and Sleep Variables between Patients with Major Depression and Healthy Controls

	MDD (n = 34)					
	TRD (n = 20)	Non-TRD (n = 14)	Healthy Control (n = 34)	P-value^a^	P-value^b^	Post-Hoc
Age (years, SD)	45.1 (10.2)	47.9 (11.1)	42.5 (8.5)	0.110	0.198	
Female (n, %)	12 (60.0)	8 (57.1)	20 (58.5)	1.000	0.986	
BMI (SD)	25.3 (4.4)	24.6 (4.8)	23.3 (4.8)	0.152	0.331	
Duration of illness	12.6 (11.1)	8.9 (9.0)	0	<0.001	<0.001	TRD, non-TRD > HC
HAMD–17	19.6 (10.3)	10.86 (7.2)	0.71 (1.2)	<0.001	<0.001	TRD > non-TRD > HC
Psychotropic medication use						
Antidepressants	16 (80)	9 (64.3)	0	<0.001	<0.001	TRD, non-TRD > HC
Antipsychotics	12 (60)	1 (7.1)	0	<0.001	<0.001	TRD > non-TRD, HC
Mood stabilizers	6 (30)	0	0	0.025	<0.001	TRD > non-TRD, HC
Cytokine levels (SD)						
Log sTNF-αR1	3.12 (0.12)	3.02 (0.11)	3.00 (0.11)	0.007	0.001	TRD > non-TRD, HC
Log CRP	3.06 (0.62)	2.88 (0.48)	2.90 (0.63)	0.552	0.598	
Log sIL–2R	2.93 (0.17)	2.81 (0.17)	2.86 (0.17)	0.563	0.089	
Log sIL–6R	4.61 (0.14)	4.59 (0.12)	4.61 (0.12)	0.733	0.782	
Log MCP–1	2.42 (0.20)	2.47 (0.18)	2.39 (0.16)	0.265	0.413	
PSQI sleep index						
Sleep quality	2.2 (0.8)	1.9 (0.8)	0.9 (0.7)	<0.001	<0.001	TRD, non-TRD > HC
Sleep latency	2.2 (1.0)	1.8 (0.9)	0.6 (0.8)	<0.001	<0.001	TRD, non-TRD > HC
Sleep duration	1.8 (1.3)	1.1 (1.1)	0.9 (1.0)	0.043	0.044	
Sleep efficiency	0.8 (1.1)	0.4 (0.6)	0.2 (0.4)	0.036	0.119	
Sleep disturbances	2.0 (0.9)	1.9 (0.5)	0.8 (0.6)	<0.001	<0.001	TRD, non-TRD > HC
Use of sleeping medications	2.4 (1.2)	1.4 (1.5)	0.0 (0.5)	<0.001	<0.001	TRD, non-TRD > HC
Daytime dysfunction	1.9 (1.0)	1.6 (0.6)	0.3 (0.5)	<0.001	<0.001	TRD, non-TRD > HC
Global scores	13.0 (5.0)	10.0 (2.4)	3.7 (2.9)	<0.001	<0.001	TRD, non-TRD > HC

Abbreviations: BMI, body mass index; CRP, C-reactive protein; HAMD-17, 17-item version of Hamilton depression rating scale; MDD, major depressive disorder; MCP-1, Monocyte chemoattractant protein-1; PSQI, Pittsburgh sleep quality index; sIL-2R, soluble interleukin-2 receptor; sIL-6R, soluble interleukin-6 receptor; sTNF-αR1, soluble tumor necrosis factor-α receptor type 1; TRD, treatment-resistant depression.

a Independent t-tests comparing MDD and HC groups.

b ANOVA comparing TRD, non-TRD and HC groups. Levene test was done to test differences of variances among groups. Welch one-way analysis of variance was performed when the homogeneity of variance was not equally distributed, and the post-hoc Games-Howell test was then performed to determine pairwise differences between groups.

Linear regression analyses adjusting for covariates showed that serum sTNF-αR1 level positively correlated with sleep latency, while serum sIL-2R level positively associated with PSQI total score among patients with MDD (Table 2). Sub-analyses stratified by disease group found positive associations between sTNF-αR1 and sleep latency in both the TRD and non-TRD group (both p < 0.05). However, after applying false discovery rate correction, the associations became statistically insignificant in each group. CRP, sIL-6R, and MCP-1 were not associated with subjective sleep quality in this study (Supplementary Table S1). Component 2 score (sleep latency) significantly correlated with component 3 score among patients with MDD (sleep duration; B = 0.419, 95% CI = 0.193–0.646, t = 3.768, R^2^ = 0.307). In addition, sex remained insignificant across all linear regression analyses between inflammation and subjective sleep quality.

**Table 2.** Correlation of sTNFaR1, sIL-2R, and PSQI Score Among Patients with Major Depression with the Adjustment of Age, Sex, BMI, HAMD-17 Total Scores, Duration of Illness, Psychotropic Medication Use, and Disease Group

	Log sTNF-αR1					
	B	95% CI	*p*	Log sIL-2R		
Sleep quality	0.017	−0.040–0.075	0.537	0.038	−0.051–0.128	0.387
Sleep latency	0.073	0.030–0.115	0.002	0.067	−0.009–0.144	0.083
Sleep duration	0.011	−0.026–0.047	0.545	0.026	−0.031–0.083	0.363
Sleep efficiency	0.006	−0.041–0.053	0.806	−0.029	−0.103–0.045	0.425
Sleep disturbances	−0.006	−0.074–0.063	0.860	0.005	−0.104–0.113	0.932
Use of sleeping medications	0.018	−0.013–0.050	0.240	0.051	0.004–0.098	0.034
Daytime dysfunction	−0.009	−0.069–0.050	0.751	0.073	−0.016–0.163	0.105
Global scores	0.010	−0.003–0.022	0.121	0.019	0.000–0.038	0.049

Abbreviations: CI, confidence interval, GLM, general linear model; BMI, body mass index; HAMD-17, 17-item version of Hamilton depression rating scale; PSQI, Pittsburgh sleep quality index; sIL-2R, soluble interleukin-2 receptor; sTNF-αR1, soluble tumor necrosis factor-α receptor type 1.

**Figure 1.**
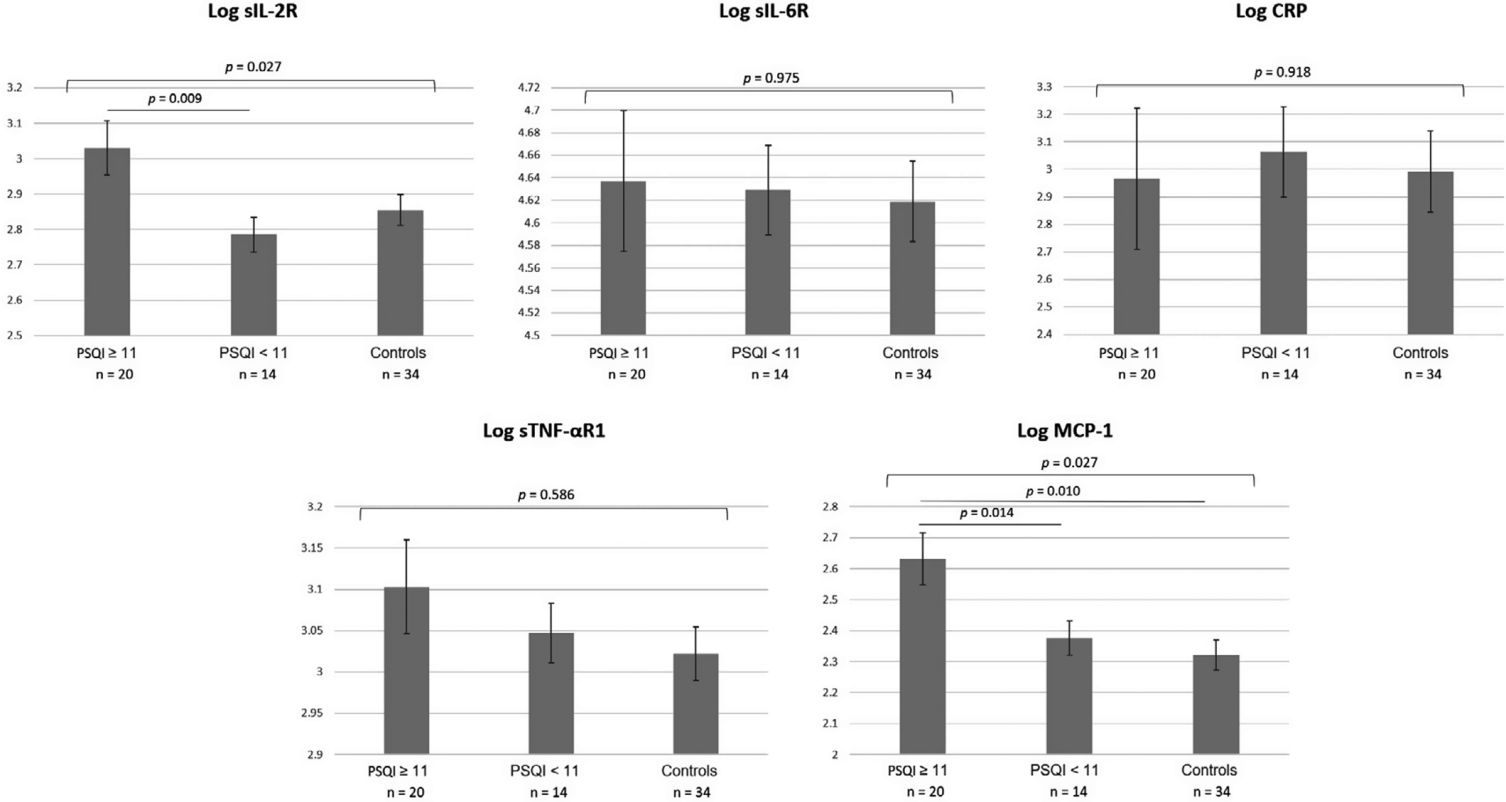
Comparison of pro-inflammatory cytokine levels among MDD patients with high (PSQI total score ≥ 11) and low (PSQI total score < 11) sleep disturbance and healthy controls. Cytokine levels were analyzed using a general linear model, adjusting for age, sex, BMI, HAMD-17 total score, duration of illness, psychotropic medication use, and disease group. Abbreviation: BMI, body mass index; CRP, C-reactive protein; HAMD-17, 17-item version of Hamilton depression rating scale; MDD, major depressive disorder; MCP-1, Monocyte chemoattractant protein-1; PSQI, Pittsburgh sleep quality index; sIL-2R, soluble interleukin-2 receptor; sIL-6R, soluble interleukin-6 receptor; sTNF-αR1, soluble tumor necrosis factor-α receptor type 1; TRD, treatment-resistant depression.

**Figure 2.**
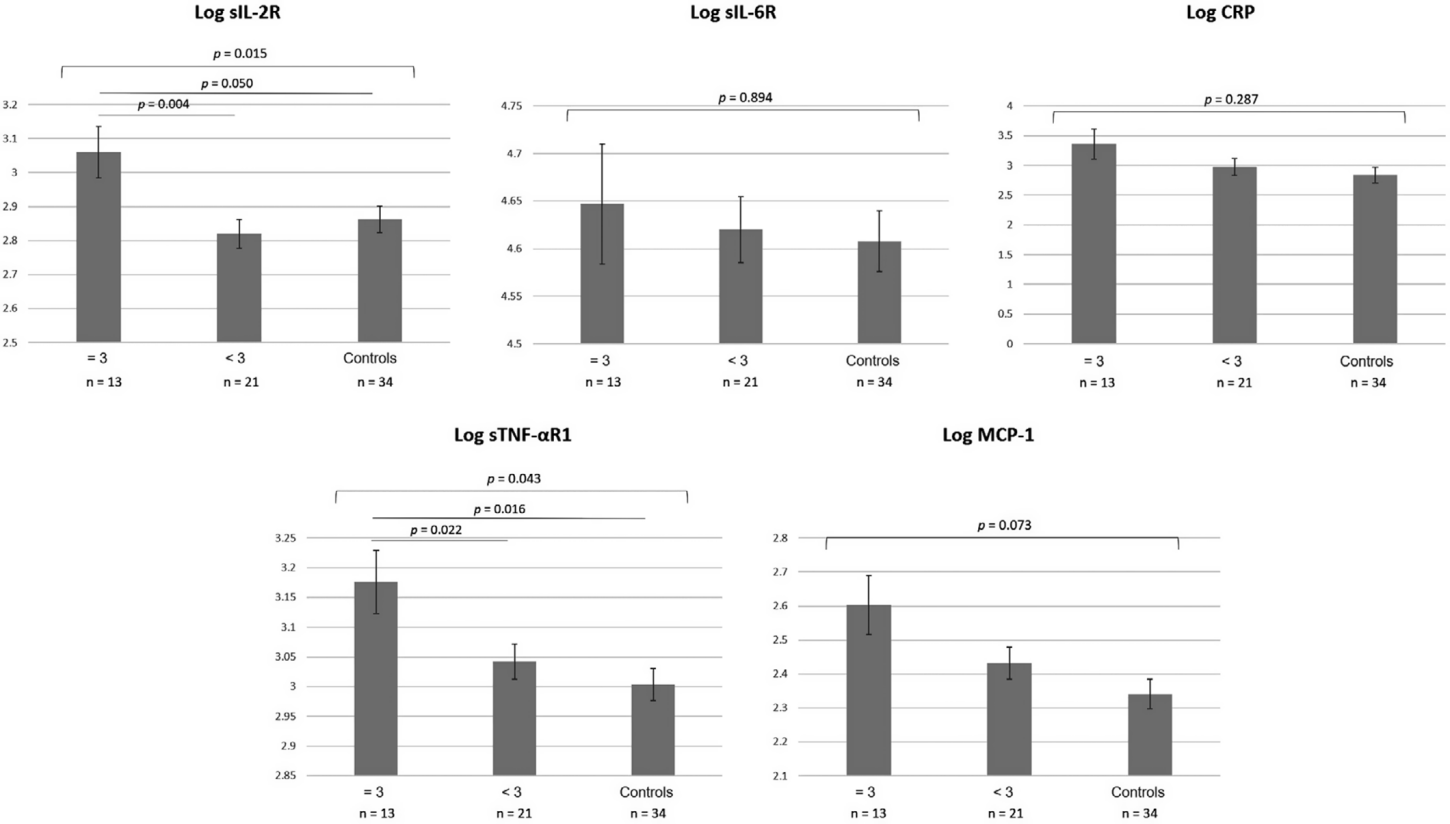
Comparison of pro-inflammatory cytokine levels among 3 groups: patients with severe sleep latency disturbance (component 2 score = 3), patients with mild-to-moderate sleep latency disturbance (component 2 score < 3), and healthy subjects. The analysis was adjusted for age, sex, BMI, HAMD-17 total scores, duration of illness, psychotropic medication use, and disease groups. Adjusted cytokine levels were estimated using a general linear model with post-hoc comparisons. Abbreviation: BMI, body mass index; CRP, C-reactive protein; HAMD-17, 17-item version of Hamilton depression rating scale; MDD, major depressive disorder; MCP-1, Monocyte chemoattractant protein-1; PSQI, Pittsburgh sleep quality index; sIL-2R, soluble interleukin-2 receptor; sIL-6R, soluble interleukin-6 receptor; sTNF-αR1, soluble tumor necrosis factor-α receptor type 1; TRD, treatment-resistant depression.

Stratified by PSQI scores, GLM analyses adjusting for covariates showed that patients with severe sleep disturbance (PSQI ≥11) had significantly higher serum levels of sIL-2R and MCP-1 than other patients (Figure 1). MDD patients with difficulty initiating sleep (component 2: sleep latency score = 3) had higher serum levels of sIL-2R and sTNF-αR1 than other patients and controls (Figure 2). Patients stratified by other component scores of PSQI did not show differences in peripheral inflammatory markers between groups.

## Discussion

The current study found that higher serum levels of sTNF-αR1 were associated with longer sleep latency across TRD and non-TRD groups. Elevated serum sIL-2R levels correlated with poorer overall sleep quality among patients with MDD. Severe sleep disturbance correlated with increased sIL-2R and MCP-1 levels, while patients with sleep initiation difficulties or sleep loss had elevated sIL-2R and sTNF-αR1 levels. Other inflammatory markers tested were not associated with subjective sleep quality in this study.

In our study, serum sTNF-αR1 level was significantly associated with sleep latency in patients with MDD, including both TRD and non-TRD subgroups. However, after applying false discovery rate correction for multiple comparisons, the associations became statistically nonsignificant. This suggests that the observed associations should be interpreted with caution, as they may be influenced by sample size and multiple testing adjustments. Given the biological plausibility of TNF-α’s role in sleep disturbances and prior evidence in the literature, future studies with larger samples are needed to validate these findings. The TNF-α system has emerged as a crucial player in sleep regulation and circadian biology, evident in both animal and human studies.^9^^,^^28^ In rodents, both acute sleep deprivation and chronic sleep restriction have been observed to elevate TNF-α expression in various brain regions, including the cortex, hippocampus, and brainstem, indicating diurnal fluctuations in TNF-α levels.^29^ In healthy individuals, acute sleep deprivation was shown to induce secretion of TNF-α, but not IL-6 or CRP.^30^ A reduction in total sleep duration (from 8 to 6 hours per night for 1 week in healthy subjects) has been associated with increased TNF-α levels the following day. In pathological conditions associated with shortened sleep duration, chronic elevations of systemic TNF-α have been observed.^31^ Our observational results in MDD correspond to previous studies that TNF-α may serve as a mediator of pathological or experimentally induced sleepiness. Conversely, TNF-α can modulate sleep, and experimental studies across species by intravenous administration or microinjections into certain brain areas have consistently shown that TNF-α enhances non-rapid eye movement sleep (NREMS) duration and induces sleepiness.^32^^,^^33^ Blockade of TNF-α has shown promise in reducing depressive symptoms in patients with increased inflammation, yet its effect on sleep in depressed patients remains underexplored. A polysomnography study indicated that TNF-α blockade with infliximab may improve sleep continuity in patients with MDD, particularly those with high inflammation levels.^34^ Additionally, levels of TNF-α messenger RNA (mRNA), sTNF-αR, and sTNF-αR mRNA in the CNS also vary with sleep propensity in a similar fashion. Irwin and colleagues demonstrated increased production of TNF-α mRNA by monocytes, notably observed the morning after sleep deprivation.^35^^,^^36^ This elevation in circulating TNF-α levels could stem from the activation of cellular TNF-α expression, potentially influencing the expression of other inflammatory cytokines within cells.^35^ Furthermore, the heightened expression of inflammatory genes following sleep deprivation is attributed to the activation of nuclear factor kappa-light-chain-enhancer of activated B cells (NF-κB) transcription control pathways, which play a pivotal role in regulating the expression of pro-inflammatory genes.^11^^,^^35^ Regarding sTNF-αR, sTNF-αR1 levels rise after sleep loss in humans, while sTNF-αR2 remains unaffected.^37^ Additionally, sleep deprivation boosts sTNF-αR1 mRNA expression.^38^ Further research is warranted to elucidate the intricate mechanisms underlying TNF-α’s role in sleep regulation and its potential therapeutic implications for sleep disorders and related conditions.

We found that serum sIL-2R level was positively associated with PSQI total score among patients with MDD, and patients with problems in sleep latency had higher serum sIL-2R levels. IL-2, primarily synthesized by helper T cells, serves as a T cell growth factor with extensive immunoregulatory functions and CNS regulatory roles.^39^ The exact nature of IL-2’s inflammatory activity remains ambiguous. While historical literature has predominantly depicted IL-2 as pro-inflammatory, recent clinical investigations have suggested its anti-inflammatory properties. Researchers have observed IL-2’s capacity to suppress inflammatory responses, leading to its classification as both pro- and anti-inflammatory.^40^^,^^41^ Limited human studies have explored the relationship between sleep and IL-2. Previous studies in humans found that sleep deprivation increased IL-2 activity, with increased IL-2 production during sleep recovery.^42^^,^^43^ Sleep deprivation may result in an upregulation of TNF-α, which in turn stimulates NF-κB and subsequently increases IL-2 production.^44^ This could explain our observation of the association between high sIL-2R levels and longer sleep latency. The sleep-inducing effects of IL-2 may be mediated through various mechanisms, including opioid receptor antagonism, induction of sleep regulatory substances such as TNF-α, and activation of neuronal nitric oxide (NO) synthase. These findings suggest a complex interplay between IL-2 and other sleep regulatory substances, highlighting the need for further research to elucidate the precise mechanisms underlying IL-2-induced sleep regulation.

This cross-sectional study suggests a potential interplay between sleep disturbance and inflammation in depression. However, the causal directionality between sleep and inflammation remains uncertain. From an evolutionary perspective, cytokines are crucial regulators of host defense against infection, and the sleep-inducing effects of cytokines, along with the activation of immune cells that stimulate cytokine production, may lead individuals with elevated inflammation levels to longer sleep durations.^45^ Experimental studies have demonstrated that cytokines can modulate sleep architecture, implying that alterations in inflammation could plausibly contribute to sleep abnormalities and subsequently elevate the risk of depression.^46^ Conversely, sleep disturbances might also contribute to increased systemic inflammation compared to periods of uninterrupted sleep.^47^ For example, sleep reduction by 2 hours per night for a week in non-depressed individuals has been shown to increase circulating TNF-α.^47^ Sleep disturbance is believed to influence the sympathetic nervous system and hypothalamic–pituitary–adrenal axis, thereby promoting inflammation. Specifically, β-adrenergic signaling has been implicated in inducing the expression of inflammatory genes, cytokine production, and systemic inflammation.^48^

We did not find sex-specific effects of subjective sleep quality on inflammatory responses. The association between sleep patterns and health outcomes seems to exhibit sex-specific differences, with recent evidence indicating that women may be more susceptible to the consequences of poor sleep, showing heightened inflammatory responses.^36^^,^^49^ However, population-based studies have yielded conflicting results, with some showing no association between poor sleep and peripheral inflammatory markers when pooling men and women, while others suggest a stronger link in women when analyzed separately.^50^ These findings underscore the need for further investigation into the sex-specific effects of sleep disturbances on inflammatory mechanisms and subsequent health outcomes.

Our study offers valuable insights into the potential underlying mechanisms of MDD and its related symptoms, namely sleep disturbance. It can inform the development of targeted interventions aimed at improving both sleep quality and inflammation, thereby potentially ameliorating the burden of depression and reducing the risk of related health complications.^51^ Chronic inflammation, linked to adverse health conditions like cardiovascular disease and diabetes, underscores the importance of investigating how poor sleep quality, inflammation, and depression interact. In addition, our team’s past research on patients with MDD found that inflammation is associated with cognitive impairment and suicidal ideation;^5^^,^^26^ this study further expands the clinical influences of inflammation on sleep disturbance in MDD.

This study has several limitations. First, the sample size was relatively small. Studies with a larger sample size are required to avoid a type II error. Second, a cross-sectional design limits causality establishment between variables, only allowing for associations to be observed. To better understand the mediating role of inflammation in the relationship between MDD incidence and sleep disturbances, longitudinal studies or experimental manipulations of sleep and/or cytokines are necessary.^52^ Third, other inflammatory markers, such as IL-1β or neutrophil-to-lymphocyte ratio, may also be relevant to the pathophysiology of depression and sleep disturbance, but were not included in this study.^53^^,^^54^ Also, inflammatory markers were measured only once in the current study, whereas repeated measurements would have provided a more robust validation of inflammatory activity. Fourth, the associations between sleep disturbance and inflammation varied by the method of sleep assessment,^12^ as polysomnography or actigraphic assessment was not utilized. Objective sleep parameters and sleep apnea were not evaluated in this study, potentially impacting the results, given the known association between obstructive sleep apnea and increased inflammatory activity, including alterations in TNF-α production.^55^ Additionally, lifestyle factors such as diet and physical activity,^56^^,^^57^ which could influence inflammation and sleep quality, were not collected in our study. Last, a considerable portion of patients were prescribed psychotropic medications in our study, particularly antidepressants, known to impact REM latency and REM sleep duration; some psychotropic medications carry anti-inflammatory effects.^58^ Cautious interpretation of our results due to potential confounding effects of medications on sleep quality and inflammation is needed,^59^ but preserving patients’ ongoing medication prevents disease relapse and exacerbation of symptoms throughout the study period and was ethically imperative.

This study elucidates the intricate relationship between sleep disturbance and inflammation among patients with MDD, particularly focusing on inflammatory markers such as sTNF-αR1 and sIL-2R. Patients with TRD exhibited higher levels of serum sTNF-αR1. Serum sTNF-αR1 levels positively correlated with sleep latency, and serum sIL-2R levels were associated with poorer overall sleep quality and specific sleep disturbances. These findings indicate the interplay between inflammation and sleep in MDD. Further longitudinal research is warranted to elucidate causal relationships, so as to inform potential therapeutic interventions targeting both domains.

## Supporting information

Huang et al. supplementary material Huang et al. supplementary material

## Data Availability

The data that support the findings of this study are available on request from the corresponding author. The data are not publicly available due to privacy or ethical restrictions.

## Supplementary material

The supplementary material for this article can be found at http://doi.org/10.1017/S1092852925000227.

## References

[1] Nutt D, Wilson S, Paterson L. Sleep disorders as core symptoms of depression. Dialogues Clin Neurosci. 2008;10(3):329–336. 18979946 10.31887/DCNS.2008.10.3/dnutt PMC3181883

[2] Steiger A, Pawlowski M. Depression and sleep. Int J Mol Sci. 2019;20(3):607. 30708948 10.3390/ijms20030607 PMC6386825

[3] Miller AH, Raison CL. The role of inflammation in depression: from evolutionary imperative to modern treatment target. Nat Rev Immunol. 2016;16(1):22–34. 26711676 10.1038/nri.2015.5 PMC5542678

[4] Capuron L, Miller AH. Immune system to brain signaling: neuropsychopharmacological implications. Pharmacol Ther. 2011;130(2):226–238. 21334376 10.1016/j.pharmthera.2011.01.014 PMC3072299

[5] Huang MH, Chen MH, Hsu JW, et al. Inflammation, cognitive dysfunction, and suicidal ideation among patients with major depression. CNS Spectr. 2022;27(6):724–730. 34423759 10.1017/S1092852921000729

[6] Suarez EC, Krishnan RR, Lewis JG. The relation of severity of depressive symptoms to monocyte-associated proinflammatory cytokines and chemokines in apparently healthy men. Psychosom Med. 2003;65(3):362–368. 12764208 10.1097/01.psy.0000035719.79068.2b

[7] Opp MR. Sleeping to fuel the immune system: mammalian sleep and resistance to parasites. BMC Evol Biol. 2009;9:8. 19134176 10.1186/1471-2148-9-8 PMC2633283

[8] Krueger JM, Obal FJ, Fang J, et al. The role of cytokines in physiological sleep regulation. Ann N Y Acad Sci. 2001;933:211–221. 12000022 10.1111/j.1749-6632.2001.tb05826.x

[9] Krueger JM. The role of cytokines in sleep regulation. Curr Pharm Des. 2008;14(32):3408–3416. 19075717 10.2174/138161208786549281 PMC2692603

[10] Capuron L, Ravaud A, Gualde N, et al. Association between immune activation and early depressive symptoms in cancer patients treated with interleukin-2-based therapy. Psychoneuroendocrinology. 2001;26(8):797–808. 11585680 10.1016/s0306-4530(01)00030-0

[11] Irwin MR, Wang M, Ribeiro D, et al. Sleep loss activates cellular inflammatory signaling. Biol Psychiatry. 2008;64(6):538–540. 18561896 10.1016/j.biopsych.2008.05.004 PMC2547406

[12] Irwin MR, Olmstead R, Carroll JE. Sleep disturbance, sleep duration, and inflammation: a systematic review and meta-analysis of cohort studies and experimental sleep deprivation. Biol Psychiatry. 2016;80(1):40–52. 26140821 10.1016/j.biopsych.2015.05.014 PMC4666828

[13] Slavish DC, Graham-Engeland JE, Engeland CG, et al. Insomnia symptoms are associated with elevated C-reactive protein in young adults. Psychol Health. 2018;33(11):1396–1415. 30358412 10.1080/08870446.2018.1500577

[14] Fernandez-Mendoza J, Baker JH, Vgontzas AN, et al. Insomnia symptoms with objective short sleep duration are associated with systemic inflammation in adolescents. Brain Behav Immun. 2017;61:110–116. 28041986 10.1016/j.bbi.2016.12.026 PMC5316336

[15] Li L, Wu C, Gan Y, et al. Insomnia and the risk of depression: a meta-analysis of prospective cohort studies. BMC Psychiatry. 2016;16(1):375. 27816065 10.1186/s12888-016-1075-3 PMC5097837

[16] Berlim MT, Turecki G. Definition, assessment, and staging of treatment-resistant refractory major depression: a review of current concepts and methods. Can J Psychiatry. 2007;52(1):46–54. 17444078 10.1177/070674370705200108

[17] Chamberlain SR, Cavanagh J, de Boer P, et al. Treatment-resistant depression and peripheral C-reactive protein. Br J Psychiatry. 2019;214(1):11–19. 29764522 10.1192/bjp.2018.66 PMC6124647

[18] Strawbridge R, Arnone D, Danese A, et al. Inflammation and clinical response to treatment in depression: a meta-analysis. Eur Neuropsychopharmacol. 2015;25(10):1532–1543. 26169573 10.1016/j.euroneuro.2015.06.007

[19] Raison CL, Rutherford RE, Woolwine BJ, et al. A randomized controlled trial of the tumor necrosis factor antagonist infliximab for treatment-resistant depression: the role of baseline inflammatory biomarkers. JAMA Psychiatry. 2013;70(1):31–41. 22945416 10.1001/2013.jamapsychiatry.4 PMC4015348

[20] Huang MH, Chen MH, Tu PC, et al. Elevated tumor necrosis factor-alpha receptor subtype 1 and the association with abnormal brain function in treatment-resistant depression. J Affect Disord. 2018;235:250–256. 29660639 10.1016/j.jad.2018.04.037

[21] Buysse DJ, Reynolds CF, 3rd, Monk TH, et al. The Pittsburgh sleep quality index: a new instrument for psychiatric practice and research. Psychiatry Res. 1989;28(2):193–213. 2748771 10.1016/0165-1781(89)90047-4

[22] Zheng Y, Wang L, Feng L, et al. Sleep quality and mental health of medical workers during the coronavirus disease 2019 pandemic. Sleep Biol Rhythms. 2021;19(2):173–180. 33456342 10.1007/s41105-020-00304-7 PMC7797025

[23] Ting EY, Yang AC, Tsai SJ. Role of interleukin-6 in depressive disorder. Int J Mol Sci. 2020;21(6): 2194. 32235786 10.3390/ijms21062194 PMC7139933

[24] Bai YM, Su TP, Li CT, et al. Comparison of pro-inflammatory cytokines among patients with bipolar disorder and unipolar depression and normal controls. Bipolar Disord. 2015;17(3):269–277. 25257835 10.1111/bdi.12259

[25] Ma K, Zhang H, Baloch Z. Pathogenetic and therapeutic applications of tumor necrosis factor-alpha (TNF-alpha) in major depressive disorder: a systematic review. Int J Mol Sci. 2016;17(5): 733. 27187381 10.3390/ijms17050733 PMC4881555

[26] Huang MH, Chan YE, Chen MH, et al. Pro-inflammatory cytokines and cognitive dysfunction among patients with bipolar disorder and major depression. Psychiatry Clin Neurosci. 2022;76(9):450–458. 35674415 10.1111/pcn.13433

[27] Deshmane SL, Kremlev S, Amini S, et al. Monocyte chemoattractant protein-1 (MCP-1): an overview. J Interferon Cytokine Res. 2009;29(6):313–326. 19441883 10.1089/jir.2008.0027 PMC2755091

[28] Rockstrom MD, Chen L, Taishi P, et al. Tumor necrosis factor alpha in sleep regulation. Sleep Med Rev. 2018;40:69–78. 29153862 10.1016/j.smrv.2017.10.005 PMC5955790

[29] Zielinski MR, McKenna JT, McCarley RW. Functions and mechanisms of sleep. AIMS Neurosci. 2016;3(1):67–104. 28413828 10.3934/Neuroscience.2016.1.67 PMC5390528

[30] Chennaoui M, Sauvet F, Drogou C, et al. Effect of one night of sleep loss on changes in tumor necrosis factor alpha (TNF-alpha) levels in healthy men. Cytokine. 2011;56(2):318–324. 21737301 10.1016/j.cyto.2011.06.002

[31] Haack M, Pollmacher T, Mullington JM. Diurnal and sleep-wake dependent variations of soluble TNF- and IL-2 receptors in healthy volunteers. Brain Behav Immun. 2004;18(4):361–367. 15157953 10.1016/j.bbi.2003.12.009

[32] Mullington J, Korth C, Hermann DM, et al. Dose-dependent effects of endotoxin on human sleep. Am J Physiol Regul Integr Comp Physiol. 2000;278(4):R947–955. 10749783 10.1152/ajpregu.2000.278.4.R947

[33] Terao A, Matsumura H, Yoneda H, et al. Enhancement of slow-wave sleep by tumor necrosis factor-alpha is mediated by cyclooxygenase-2 in rats. Neuroreport. 1998;9(17):3791–3796. 9875706 10.1097/00001756-199812010-00005

[34] Weinberger JF, Raison CL, Rye DB, et al. Inhibition of tumor necrosis factor improves sleep continuity in patients with treatment resistant depression and high inflammation. Brain Behav Immun. 2015;47:193–200. 25529904 10.1016/j.bbi.2014.12.016 PMC4468009

[35] Irwin MR, Wang M, Campomayor CO, et al. Sleep deprivation and activation of morning levels of cellular and genomic markers of inflammation. Arch Intern Med. 2006;166(16):1756–1762. 16983055 10.1001/archinte.166.16.1756

[36] Irwin MR, Carrillo C, Olmstead R. Sleep loss activates cellular markers of inflammation: sex differences. Brain Behav Immun. 2010;24(1):54–57. 19520155 10.1016/j.bbi.2009.06.001 PMC2787978

[37] Shearer WT, Reuben JM, Mullington JM, et al. Soluble TNF-alpha receptor 1 and IL-6 plasma levels in humans subjected to the sleep deprivation model of spaceflight. J Allergy Clin Immunol. 2001;107(1):165–170. 11150007 10.1067/mai.2001.112270

[38] Taishi P, Gardi J, Chen Z, et al. Sleep deprivation increases the expression of TNF alpha mRNA and TNF 55kD receptor mRNA in rat brain. Physiologist. 1999;42:A4.

[39] Malek TR. The biology of interleukin-2. Annu Rev Immunol. 2008;26:453–479. 18062768 10.1146/annurev.immunol.26.021607.090357

[40] Boerrigter D, Weickert TW, Lenroot R, et al. Using blood cytokine measures to define high inflammatory biotype of schizophrenia and schizoaffective disorder. J Neuroinflammation. 2017;14(1):188. 28923068 10.1186/s12974-017-0962-y PMC5604300

[41] Lan RY, Selmi C, Gershwin ME. The regulatory, inflammatory, and T cell programming roles of interleukin-2 (IL-2). J Autoimmun. 2008;31(1):7–12. 18442895 10.1016/j.jaut.2008.03.002

[42] Born J, Lange T, Hansen K, et al. Effects of sleep and circadian rhythm on human circulating immune cells. J Immunol. 1997;158(9):4454–4464. 9127011

[43] Irwin M, McClintick J, Costlow C, et al. Partial night sleep deprivation reduces natural killer and cellular immune responses in humans. FASEB J. 1996;10(5):643–653. 8621064 10.1096/fasebj.10.5.8621064

[44] Krueger JM, Majde JA, Obal F. Sleep in host defense. Brain Behav Immun. 2003;17(Suppl 1):S41–S47. 12615185 10.1016/s0889-1591(02)00065-x

[45] Vgontzas AN, Papanicolaou DA, Bixler EO, et al. Elevation of plasma cytokines in disorders of excessive daytime sleepiness: role of sleep disturbance and obesity. J Clin Endocrinol Metab. 1997;82(5):1313–1316. 9141509 10.1210/jcem.82.5.3950

[46] Haack M, Schuld A, Kraus T, et al. Effects of sleep on endotoxin-induced host responses in healthy men. Psychosom Med. 2001;63(4):568–578. 11485110 10.1097/00006842-200107000-00008

[47] Vgontzas AN, Zoumakis E, Bixler EO, et al. Adverse effects of modest sleep restriction on sleepiness, performance, and inflammatory cytokines. J Clin Endocrinol Metab. 2004;89(5):2119–2126. 15126529 10.1210/jc.2003-031562

[48] Hall MH, Smagula SF, Boudreau RM, et al. Association between sleep duration and mortality is mediated by markers of inflammation and health in older adults: the health, aging and body composition study. Sleep. 2015;38(2):189–195. 25348127 10.5665/sleep.4394 PMC4288599

[49] Suarez EC. Self-reported symptoms of sleep disturbance and inflammation, coagulation, insulin resistance and psychosocial distress: evidence for gender disparity. Brain Behav Immun. 2008;22(6):960–968. 18328671 10.1016/j.bbi.2008.01.011 PMC3652592

[50] Taheri S, Austin D, Lin L, et al. Correlates of serum C-reactive protein (CRP)--no association with sleep duration or sleep disordered breathing. Sleep. 2007;30(8):991–996. 17702268 10.1093/sleep/30.8.991 PMC1978379

[51] Mallon L, Broman JE, Hetta J. Sleep complaints predict coronary artery disease mortality in males: a 12-year follow-up study of a middle-aged Swedish population. J Intern Med. 2002;251(3):207–216. 11886479 10.1046/j.1365-2796.2002.00941.x

[52] Prather AA, Rabinovitz M, Pollock BG, et al. Cytokine-induced depression during IFN-alpha treatment: the role of IL-6 and sleep quality. Brain Behav Immun. 2009;23(8):1109–1116. 19615438 10.1016/j.bbi.2009.07.001 PMC2783448

[53] Norman GJ, Karelina K, Zhang N, et al. Stress and IL-1beta contribute to the development of depressive-like behavior following peripheral nerve injury. Mol Psychiatry. 2010;15(4):404–414. 19773812 10.1038/mp.2009.91 PMC5214062

[54] Fusar-Poli L, Natale A, Amerio A, et al. Neutrophil-to-lymphocyte, platelet-to-lymphocyte and monocyte-to-lymphocyte ratio in bipolar disorder. Brain Sci. 2021;11(1): 58. 33418881 10.3390/brainsci11010058 PMC7825034

[55] Tamaki S, Yamauchi M, Fukuoka A, et al. Production of inflammatory mediators by monocytes in patients with obstructive sleep apnea syndrome. Intern Med. 2009;48(15):1255–1262. 19652426 10.2169/internalmedicine.48.2366

[56] Kiecolt-Glaser JK. Stress, food, and inflammation: psychoneuroimmunology and nutrition at the cutting edge. Psychosom Med. 2010;72(4):365–369. 20410248 10.1097/PSY.0b013e3181dbf489 PMC2868080

[57] Gleeson M, Bishop NC, Stensel DJ, et al. The anti-inflammatory effects of exercise: mechanisms and implications for the prevention and treatment of disease. Nat Rev Immunol. 2011;11(9):607–615. 21818123 10.1038/nri3041

[58] Beurel E, Jope RS. Inflammation and lithium: clues to mechanisms contributing to suicide-linked traits. Transl Psychiatry. 2014;4(12):e488. 25514751 10.1038/tp.2014.129 PMC4270310

[59] Pompili M, Venturini P, Palermo M, et al. Mood disorders medications: predictors of nonadherence—review of the current literature. Expert Rev Neurother. 2013;13(7):809–825. 23898852 10.1586/14737175.2013.811976

