# Supplementary material for: Associations between subjective sleep quality and inflammatory markers in patients with treatment-resistant depression
Source: CNS Spectr. 2025 Apr 11;30(1):e60. doi: 10.1017/S1092852925000227 (PMC13064773; doi:10.1017/S1092852925000227)
Supplement: Huang et al. supplementary material [file S1092852925000227sup001.docx]

Supplementary Table 1. Correlation of proinflammatory cytokines and PSQI score among patients with major depression with the adjustment of age, sex, BMI, HAMD-17 total scores, duration of illness, psychotropic medication use, and disease group

|  | Log CRP | | | Log sIL-6R | | | Log MCP-1 | | |
| --- | --- | --- | --- | --- | --- | --- | --- | --- | --- |
|  | B | 95% CI | *p* | B | 95% CI | *p* | B | 95% CI | *p* |
| sleep quality | -0.057 | -0.329 – 0.214 | 0.668 | -0.002 | -0.076 – 0.072 | 0.958 | 0.026 | -0.076 – 0.128 | 0.609 |
| sleep latency | 0.163 | -0.071 – 0.397 | 0.164 | -0.026 | -0.091 – 0.039 | 0.427 | 0.081 | -0.004 – 0.166 | 0.062 |
| sleep duration | 0.005 | -0.169 – 0.179 | 0.954 | 0.004 | -0.043 – 0.052 | 0.847 | -0.003 | -0.069 – 0.062 | 0.915 |
| sleep efficiency | -0.003 | -0.226 – 0.221 | 0.979 | -0.018 | -0.078 – 0.042 | 0.538 | -0.042 | -0.124 – 0.040 | 0.306 |
| sleep disturbances | 0.008 | -0.316 – 0.332 | 0.959 | -0.017 | -0.105 – 0.070 | 0.687 | 0.043 | -0.077 – 0.164 | 0.467 |
| use of sleeping medications | 0.036 | -0.117 – 0.189 | 0.634 | -0.017 | -0.058 – 0.024 | 0.392 | 0.057 | 0.004 – 0.110 | 0.037 |
| daytime dysfunction | -0.070 | -0.352 – 0.211 | 0.611 | -0.022 | -0.098 – 0.054 | 0.560 | 0.008 | -0.98 – 0.114 | 0.883 |
| global scores | 0.011 | -0.051 – 0.072 | 0.724 | -0.007 | -0.023 – 0.009 | 0.388 | 0.014 | -0.009 – 0.036 | 0.213 |

BMI: body mass index; CI: confidence interval; CRP: C-reactive protein; GLM: general linear model; HAMD-17: 17-item version of Hamilton depression rating scale; MCP-1: Monocyte chemoattractant protein-1; PSQI: Pittsburgh sleep quality index; sIL-6R: soluble interleukin-6 receptor
